# Respirasome Persistence Without Efficient Electron Channeling in Cardiac Mitochondria From an Animal Model of Hypertension

**DOI:** 10.1111/nyas.70440

**Published:** 2026-10-30

**Authors:** Patrycja Anna Glogowski, Cristina Algieri, Antonia Cugliari, Maria Cotugno, Fabiana Trombetti, Micaela Fabbri, Arrigo Francesco Giuseppe Cicero, Speranza Rubattu, Salvatore Nesci

**Affiliations:** ^1^ Cardiovascular Medicine Unit IRCCS Azienda Ospedaliero‐Universitaria di Bologna Bologna Italy; ^2^ Department of Life Sciences, Health and Health Professions Link University Rome Italy; ^3^ Department of Veterinary Medical Sciences University of Bologna Ozzano dell'Emilia Italy; ^4^ IRCCS Neuromed Pozzilli Italy; ^5^ Medical and Surgical Sciences Department Hypertension and Cardiovascular Risk Research Center Alma Mater Studiorum—University of Bologna, Sant'Orsola—Malpighi Hospital Bologna Italy; ^6^ Clinical and Molecular Department Sapienza University of Rome Rome Italy

**Keywords:** cardiac mitochondria, cytochrome *c* oxidase, electron flux control, respiratory supercomplexes, spontaneously hypertensive rats

## Abstract

Cardiac mitochondrial dysfunction contributes to hypertensive remodeling, but whether respiratory supercomplex organization preserves electron transfer efficiency during disease progression remains unresolved. Here, we examined the structural and functional organization of the respiratory complexes in cardiac mitochondria from stroke‐resistant and stroke‐prone spontaneously hypertensive rats exposed to normal or Japanese diet regimens. In both models, supramolecular analyses revealed the persistence of N‐respirasome and Q‐respirasome assemblies, indicating that major respiratory supercomplex species are maintained during sustained hypertension and in the pre‐stroke state. However, metabolic flux control analysis consistently showed that cytochrome *c* oxidase or Complex IV exerted only limited control over respiratory flux, with control coefficients below those expected for tightly channeled nicotinamide adenine dinucleotide (NADH)‐dependent electron transfer. Thus, structural preservation of respirasomes was not matched by evidence of kinetically optimized coupling. These findings uncouple supercomplex detectability from functional electron channeling and suggest that, in the hypertensive heart, respiratory performance is constrained by qualitative defects in supercomplex organization rather than by their overt loss.

## 1 Introduction

Stroke represents a growing global health challenge, with the World Stroke Organization/Lancet Neurology Commission projecting a 50% increase in stroke‐related deaths between 2020 and 2050, from 6.6 to 9.7 million annually [1]. This burden will disproportionately affect low‐ and middle‐income countries, which already account for the most stroke prevalence, mortality, and disability‐adjusted life‐years lost. Over the past three decades, stroke‐related death and disability have nearly doubled, maintaining stroke as the second leading cause of death and the third leading cause of combined death and disability worldwide. Among stroke survivors in the United States, prolonged sedentary behavior increases mortality risk, whereas recommended physical activity appears protective [2, 3].

To investigate the etiopathogenesis of stroke, several animal models have been developed, among which the stroke‐prone (SP) spontaneously hypertensive rat (SHR) is one of the most widely used [4]. The stroke‐prone spontaneously hypertensive rats (SHR‐SP) and stroke‐resistant spontaneously hypertensive rats (SHR‐SR) exhibit distinct responses to different dietary regimens. Meanwhile, SHR‐SR does not develop a stroke phenotype under any conditions; SHR‐SP exhibits accelerated stroke susceptibility when subjected to a high‐salt diet, also known as Japanese diet (JD) [5]. Comprehensive genomic analyses have revealed that the SHR‐SP strain harbors genetic determinants of stroke susceptibility, with particular emphasis on the STR1 quantitative trait locus located on rat chromosome [5]. Within this locus, comparative differential expression analyses between JD‐fed SHR‐SR and SHR‐SP rats identified nicotinamide adenine dinucleotide (NADH) dehydrogenase [ubiquinone] 1, subcomplex unknown, 2 (*Ndufc2*) as a key dysregulated gene [5]. *Ndufc2* encodes a structural subunit of mitochondrial Complex I (CI), which is essential for electron transport chain (ETC) function, as well as for CI assembly and catalytic activity, thereby underscoring a central role for mitochondrial dysfunction in stroke pathogenesis [5]. Importantly, altered NDUFC2 expression has also been associated with stroke in human studies, supporting both the translational relevance of the SHR‐SP model and the contribution of ETC impairment to cerebrovascular disease [5].

Multiple lines of evidence implicate mitochondrial dysfunction in the pathogenesis of hypertension; individuals affected by primary mitochondrial disorders exhibit a higher prevalence of hypertension, and comparative analyses of mitochondrial DNA in hypertensive versus normotensive populations have identified several variants associated with cardiovascular complications [6, 7]. Notwithstanding these observations, the molecular mechanisms underlying hypertension‐driven end‐organ damage remain incompletely understood and represent a critical unmet clinical need.

The ETC is arranged as an interconnected network of multiprotein complexes that catalyze the oxidation of reduced NADH via CI, Complex III (CIII), and Complex IV (CIV), and reduced flavin adenine dinucleotide via Complex II, coupling electron flow to proton translocation across the inner mitochondrial membrane, generating an electrochemical gradient that ultimately drives ATP synthesis by Complex V (CV) through the process of oxidative phosphorylation [8]. Structural evidence has revived the concept of the classical solid‐state model, which posits that individual respiratory complexes are organized into supramolecular assemblies, or supercomplexes (SCs). This perspective is supported primarily by consistent and reproducible observations of co‐migration of different respiratory complexes in native conditions (BN‐PAGE) and denaturing conditions (SDS–PAGE) [9]. SCs are assembled from the proton‐pumping Complexes I, III, and IV in diverse stoichiometries, including CI + CIII_2_, CIII_2_ + CIV, and larger assemblies (CI + CIII_2_ + CIV*_n_*), collectively termed “respirasome” for their ability to catalyze NADH‐O_2_ oxidoreduction in vitro. The precise physiological roles remain debated; respirasomes, particularly the most abundant CI + CIII_2_ + CIV form, have been extensively characterized structurally and biochemically, providing insights into electron transfer, proton pumping, and regulatory mechanisms [10, 11]. Although the SCs' organization could be biologically advantageous, due to enhancing the efficiency of oxidative phosphorylation via substrate channeling while limiting the production of reactive oxygen species [12, 13], there are also other possibilities.

Recently, it has been demonstrated that a portion of CIV assembles into functional SCs, facilitating substrate channeling of cytochrome *c* (cyt *c*). Specifically, in the presence of SCAF1, a CIV subunit, which promotes CIV incorporation into SCs, a substantial fraction of CIV activity is not engaged by glucose‐driven respiration [14]. In isolated liver mitochondria, it was further shown that CIV within SCs exists in three distinct functional forms: One exclusively receiving electrons from NADH oxidation, known as the N‐respirasome, in the assembly of CI + CIII_2_ + CIV; another receiving electrons from FAD‐dependent enzymes to reduce coenzyme Q, forming the CIII_2_ + CIV SC or Q‐respirasome [15]. Conversely, when CIV is permanently prevented from assembling into supercomplexes through SCAF1 deletion, the maximal respiratory activity of intact cells under glucose‐rich conditions matches the maximal respiration achieved when electrons are delivered directly to CIV. In this scenario, with all CIV in the free form and electron transfer occurring via diffusion of the cyt *c* pool, these experiments indicate that the cyt *c* may mediate electron transfer by an efficient way [16, 17].

Our study represents the first comprehensive attempt to compare the function and assembly of mitochondrial respiratory complexes in the heart tissues of SHR‐SR and SHR‐SP rats subjected to either a normosodic diet (ND) or JD. By investigating both the kinetic performance of the ETC and its supramolecular organization, this work provides novel insights into the mechanistic basis of mitochondrial dysfunction in stroke‐prone versus stroke‐resistant phenotypes.

## 2 Methods

### 2.1 Chemicals

Antimycin A, NADH, ascorbate, and cyt *c* were obtained from Sigma–Aldrich (Milan, Italy). *N*,*N*,*N*′,*N*′‐tetramethyl‐*p*‐phenylenediamine dihydrochloride (TMPD) was purchased from Merck (Darmstadt, Germany). Quartz double‐distilled water was used for all reagent solutions.

### 2.2 Animal Model

Sixteen 6‐week‐old SHR‐SR and 16 age‐matched SHR‐SP were housed at the animal facility of the Neuromed Institute (Pozzilli, Italy) and randomly assigned to receive either an ND (*n* = 8 per strain) or a JD (*n* = 8 per strain) for 4 weeks. Animals were subsequently sacrificed according to a standard protocol [5]. The ND (containing 22% protein, 2.7 mg/g Na^+^, 7.4 mg/g K^+^, 0.05 mg/g methionine) and the JD (containing 17.5% protein, 3.7 mg/g Na^+^, 6.3 mg/g K^+^, 0.03 mg/g methionine, with the addition of 1% NaCl in the drinking water) have been reported previously by Bencivenni et al. [18]; both ND and JD were provided by Laboratorio Dottori Piccioni (Milan, Italy). To capture the extreme phenotypes of stroke resistance and susceptibility, an additional cohort of SHR‐SP rats (*n* = 4) was maintained on the JD until signs of stroke, which occurred after 6–8 weeks of dietary treatment, whereas a cohort of SHR‐SR rats (*n* = 4) continued JD feeding until week 10 without developing stroke. Hearts were collected immediately after sacrifice, snap‐frozen in liquid nitrogen, and stored at −80°C for molecular analyses. Animals were housed two to three per cage under controlled light, temperature, and humidity conditions. Body weight, systolic blood pressure (measured by tail‐cuff sphygmomanometer), proteinuria, and neurological signs of stroke, including limb paresis or hemiparesis, epileptic seizures, and sudden death, were monitored throughout the study. All animal studies were performed in accordance with approved protocols by the IRCCS Neuromed Animal Care Review Board and the Istituto Superiore di Sanità (ISS permit number: 1086/2020) and were conducted according to EU Directive 2010/63/EU for animal experiments. The study is reported in accordance with the ARRIVE guidelines (https://arriveguidelines.org).

### 2.3 Preparation of Rat Heart Mitochondria

Heart rat mitochondria were isolated as described by Algieri et al. [19]; the protocol was adapted to the rat species with some modifications. Briefly, a pool of three to four thawed rat hearts was initially weighed and gently dried on blotting paper. Then it was finely chopped into small pieces with scissors and homogenized by Ultra Turrax T25 in ice‐cold Tris–HCl buffer (medium B) consisting of 0.25 M sucrose, 10 mM Tris, 1 mM EDTA (free acid), and 0.5 mg/mL BSA fatty acid free, pH 7.4 with HCl at a ratio of 10 mL medium B per 1 g of tissue. The homogenate was centrifuged to remove nuclei and plasma membrane fragments using a Sorvall RC2‐B centrifuge equipped with an SS‐34 rotor at 3000 rpm (1076 × *g*_max_) for 5 min at 4°C. The pellet was re‐homogenized and centrifuged under the same conditions. The pooled supernatant was filtered through four layers of cotton gauze and then centrifuged at 10,000 rpm (11,952 × *g*_max_) for 10 min at 4°C. The pellet was resuspended with medium A consisting of 0.25 M sucrose, 10 mM Tris(hydroxymethyl)‐aminomethane (Tris), and pH 7.4 with HCl and recentrifuged under the same conditions. Finally, the pellet was resuspended by gentle stirring using a Teflon Potter Elvehjem homogenizer in a small volume of medium A. All steps were carried out at 0–4°C [20]. The mitochondrial protein determination was performed with the colorimetric method described by Bradford using the Bio‐Rad Protein Assay kit II with BSA as standard [21]. Then, the mitochondrial aliquots were stored in liquid nitrogen.

### 2.4 Metabolic Flux Control Analysis

Flux control coefficients and threshold plots were obtained by titrating the whole respiratory chain activity, measured by NADH‐O_2_ oxidoreductase activity, and the single‐step activity with the inhibitor KCN for CIV, measured by TMPD/ASC‐O_2_ oxidoreductase activity. Moreover, the effects on flux control coefficients and thresholds were studied by addition of exogenous cyt *c* (5 µM) into the reaction, assaying both NADH‐O_2_ oxidoreductase activity and TMPD/ASC‐O_2_ oxidoreductase activity with the same KCN inhibition steps as outlined above [12, 22]. To measure mitochondrial respiratory activities, the oxygen consumption rates were polarographically evaluated by a Clark‐type electrode using a thermostatted Oxytherm System (Hansatech Instruments) equipped with a 1 mL polarographic chamber. The reaction mixture (120 mM KCl, 10 mM Tris–HCl buffer pH 7.2) was maintained at 37°C and continuously stirred, contained 0.25 mg mitochondrial protein for both SHR groups. Evaluation of NADH‐O_2_ oxidoreductase activity was performed in the presence of different concentrations of KCN using 75 µM NADH substrate. Similarly, TMPD/ASC‐O_2_ oxidoreductase activity by CIV was evaluated in the presence of 0.5 mM TMPD + 2 mM ASC by assaying cyt *c* oxidation in the presence of 1 µg/mL antimycin A. The KCN concentrations for the titration curves ranged from 0 to 2 mM, depending on the reactivity of the sample group threshold plots that were derived from titration curves by plotting the relative rate of the global respiratory activity (NADH‐O_2_ oxidoreductase) as a function of the percentage inhibition of the single‐step activity of Complex IV (TMPD/ascorbate‐O_2_ oxidoreductase), determined at identical inhibitor concentrations [12]. Control coefficients (*C_i_*) were calculated as the ratio between the slope of the inhibition curve of the global flux (*J*) and the slope of the inhibition curve of the Complex IV (*v_i_*) [12]:

$$C_{i}=\frac{{\left(dJ/dI\right)}_{I=0}}{{\left(dv_{i}/dI\right)}_{I=0}}$$

We next considered the potential biological relevance of this predominantly monomeric CIV pool in terms of the excess (reserve) capacity of the terminal oxidase, following the framework proposed by Gnaiger et al. [23]. Excess capacity was calculated, for each experimental group, as the ratio between the maximal, uninhibited (KCN = 0 mM) TMPD/ascorbate‐O_2_ oxidoreductase activity ($J_{O_{2}}$, CIV), reflecting the catalytic capacity of CIV alone, and the maximal NADH‐O_2_ oxidoreductase activity ($J_{O_{2}}$, NADH), reflecting the flux actually sustained by the upstream CI–CIII segment under the same conditions:

$$\mathrm{Excess}\;\mathrm{capacity}=\frac{J_{O_{2},\mathrm{CIV}}\left(\frac{\mathrm{TMPD}}{\mathrm{ASC}}-O_{2}\right)}{J_{O_{2},\mathrm{NADH}}\left(\mathrm{NADH}-O_{2}\right)}$$

### 2.5 Proteomic Analysis

Two‐dimensional proteomic analysis was performed using the isolated mitochondrial fraction stored in liquid nitrogen, as described by Cugliari et al. [24]. Frozen aliquots were thawed and centrifuged using a TJ‐25 Beckman Coulter centrifuge (rotor A‐15) at 11,000 × *g* for 15 min at 4°C. The resulting pellet was resuspended in resuspension buffer (50 mM NaCl, 50 mM imidazole, and 5 mM 6‐aminohexanoic acid, pH 7.0) to obtain a final protein concentration of 3.35 mg/mL. For the detergent titration, 10 µg of protein was incubated with increasing amounts of digitonin at detergent‐to‐protein ratios of 10, 8, 6, and 4 g/g. Optimal solubilization was achieved at a ratio of 4 g/g. Samples were incubated on ice for 20 min and subsequently centrifuged at 20,000 × *g* for 20 min at 4°C. An aliquot of 5 µL of the supernatant was used to determine protein concentration by the Bradford assay [21]. The remaining supernatant was supplemented with 11% glycerol, 5% Coomassie G‐250 (with a Coomassie‐to‐detergent ratio of 1:10, mg/mg), and water and then loaded into the wells of precast 10‐well NativePAGE 3%–12% Bis‐Tris mini gels (1.0 mm thickness; Thermo Fisher Scientific). A volume of 7 µL of NativeMark Unstained Protein Standard (Thermo Fisher Scientific) was loaded per gel. First‐dimension electrophoresis was performed under native conditions using a 3%–12% gradient blue native gel. Briefly, the first third of the BN‐PAGE run was performed at a constant 70 V using a dark blue cathode buffer, after which the buffer was switched to light blue cathode buffer and the gel run at 150 V until completion. The gel was then washed with MilliQ water and imaged on the ChemiDoc system. A selected gel strip from the unfixed gel was subjected to sequential chemical treatments to achieve denaturing conditions. First, the strip was incubated for 30 min at 37°C in a reducing buffer containing 4× NuPAGE LDS buffer, MilliQ water, and 50 mM 2‐mercaptoethanol. This buffer was replaced with alkylation buffer (4× NuPAGE LDS Buffer, 54.32 mM *N*,*N*‐dimethylacrylamide and MilliQ water) for 30 min and then with quenching buffer (4× NuPAGE LDS Buffer, NuPAGE sample reducing agent 10×, 19.2% ethanol, and MilliQ water) for 30 min. Then, the gel was washed with MilliQ water prior to being placed at the top of a NuPAGE 4%–12%, Bis‐Tris, 1.0–1.5 mm, Mini Protein Gels. Five µL of Biotinylated Protein Ladder (81851, Cell Signaling Technology) was placed in the control well. The SDS–PAGE gel was run at 100 V constant for 15 min, then at 150 V constant until the end of the run. The gel was incubated for 30 min with 1× Tris‐glycine and then transferred to a 0.2‐µm nitrocellulose membrane (Trans‐Blot Turbo Mini 0.2 µm Nitrocellulose Transfer Packs, BioRAD) using the mixed molecular weight 1.3°–25 V–7′ protocol of a Trans‐Blot Turbo Transfer System (BioRAD). The membrane was blocked for 1 h with 5% milk in 1× PBS containing 0.1% Tween 20. CI, CIII, and CIV were detected using the following primary antibodies: for CI, 1:1000 diluted mouse anti‐NDUFA9 monoclonal antibody (clone: 20C11B11B11, 459100, Thermofisher); for CIII, 1:1000 diluted mouse anti‐UQCRFS1 monoclonal antibody (clone: OTI4H8, MA5‐27471, Thermofisher); and for CIV, 1:1000 diluted mouse anti‐MTCO1 monoclonal antibody (clone: E7A11, bsm‐60242M, CliniSciences). All the antibodies were diluted in TBS‐Tween 20, consisting of 150 mM NaCl, 50 mM Tris–HCl, and 1% Tween 20, and were incubated overnight at 4°C. Following incubation with goat anti‐mouse IgG (H + L) cross‐adsorbed secondary antibody diluted 1:10,000 (G‐21040, Thermofisher) together with anti‐biotin, HRP‐linked antibody (7075, Cell Signaling Technology) diluted 1:1000, the blot was imaged on the ChemiDoc system following 5 min incubation with ECL (Clarity Western ECL substrate, BioRad). The 2D gel spots obtained after western blotting analysis of the subunits of the respiratory complexes were detected and quantified using ImageLab software [24].

### 2.6 Data Analysis and Statistics

Data are presented as mean ± standard deviation (SD) for each experimental group. Statistical comparisons among groups were performed using one‐way analysis of variance (ANOVA), followed by the Bonferroni post hoc test. A *p* value < 0.05 was considered statistically significant. All analyses were conducted using GraphPad Prism software (version 5.01; GraphPad Software Inc., La Jolla, CA, USA).

For the analysis of CIV excess capacity and CIV/CI relative stoichiometry, the SD of ratios between independently measured activities was estimated by standard error propagation:

$$SD_{\mathrm{ratio}}=\mathrm{Ratio}\times \sqrt{{\left(\frac{SD_{a}}{\mathrm{Mea}n_{a}}\right)}^{2}+{\left(\frac{SD_{b}}{\mathrm{Mea}n_{b}}\right)}^{2}}$$

applied whenever the two activities being compared were determined in independent sets of measurements.

## 3 Results

### 3.1 Clinical Parameters Remained Unchanged Between Normosodic and JD Groups in SHR‐SR and SHR‐SP Rats During the Pre‐Stroke Observation Period

SHR‑SR rats were maintained on either an ND or JD for up to 10 weeks, providing a longitudinal model of sustained hypertension in the absence of cerebrovascular events (Figure 1A). Throughout the experimental period, clinical parameters, including systolic blood pressure, body weight, and proteinuria, were monitored at predefined time points. No overt neurological deficits or behavioral abnormalities were detected during the study. Quantitative analysis showed that systolic blood pressure levels, body weight trajectories, and proteinuria values at 4, 6, and 10 weeks did not differ significantly between ND‑ and JD‑fed SHR‑SR rats (Figure 1A).

**FIGURE 1:**
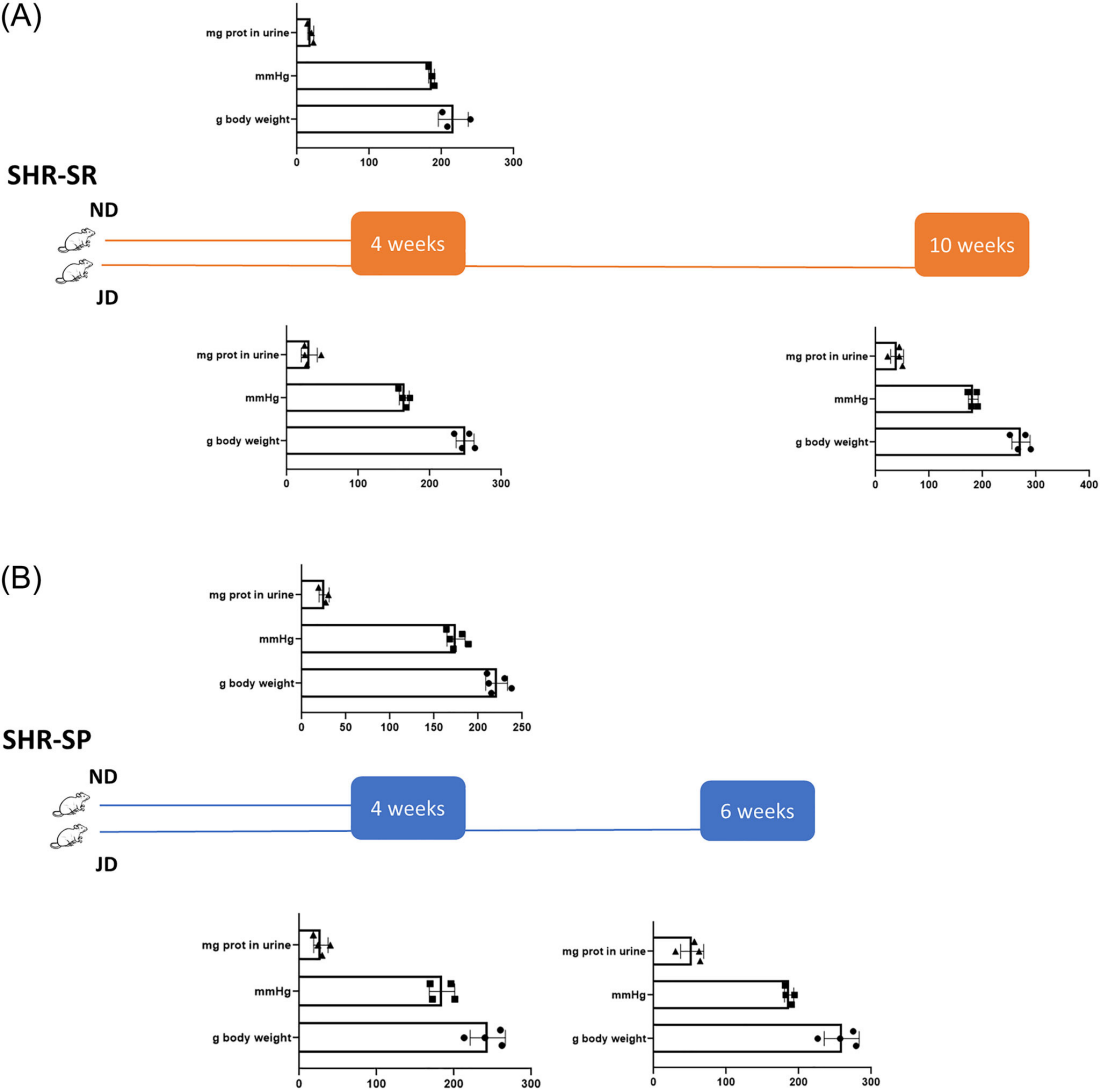
Experimental design and longitudinal assessment of clinical parameters in SHR‐SR and SHR‐SP rats. Schematic representation of the experimental protocols and clinical monitoring performed in stroke‐resistant spontaneously hypertensive rats (SHR‐SR) and stroke‐prone spontaneously hypertensive rats (SHR‐SP) fed either a normosodic diet (ND) or a Japanese diet (JD). (A) SHR‐SR rats were maintained on ND or JD for up to 10 weeks as a model of sustained hypertension in the absence of cerebrovascular events. Systolic blood pressure, body weight, and proteinuria were measured at the indicated time points (4 and 10 weeks). (B) SHR‐SP rats were maintained on ND or JD for 4 weeks to define the pre‐stroke stage, and cardiac tissues were collected before the onset of overt cerebrovascular events. A separate SHR‐SP cohort was maintained on JD for 6–8 weeks, corresponding to the time window in which the stroke phenotype typically emerged. Clinical monitoring showed systolic blood pressure, body weight, or proteinuria at matched time points.

In SHR‑SP rats, ND, or JD feeding was carried out for 4 weeks to define the pre‑stroke stage, at which time cardiac tissues were collected prior to the onset of overt cerebrovascular events (Figure 1B). A separate cohort of SHR‑SP rats was maintained on JD for 6–8 weeks, corresponding to the temporal window in which the stroke phenotype typically emerges (Figure 1B). Continuous clinical surveillance revealed stable general health conditions up to the time of sacrifice, with no significant differences in monitored parameters attributable to diet at the 4‑week time point.

Across both the SHR‑SR and SHR‑SP rat strains, longitudinal assessment demonstrated that systolic blood pressure and proteinuria levels remained comparable between ND and JD groups at matched experimental durations (4, 6, and 10 weeks, as applicable), with no statistically significant differences detected (Figure 1A,B). Body weight progression similarly showed no diet‑dependent variation within each strain. These findings confirmed that dietary intervention under the applied conditions did not differentially influence the measured clinical parameters during the observation period.

### 3.2 Limited Flux Control by Cyt *c* Oxidase Over Aerobic NADH Oxidation in SHR‐SR Heart Mitochondria

Flux control analyses and proteomic studies were performed on the function and assembly of respiratory complexes in isolated heart mitochondria from SHR‐SR rats maintained on either an ND or a JD, with the aim of assessing both the kinetic performance of the ETC and its supramolecular organization into respiratory SCs. ETC functionality was examined by metabolic flux control analysis to determine the flux *C_i_* of complexes participating in aerobic NADH oxidation under the control of Cyt *c* oxidase (CIV). *C_i_* values were calculated from the ratio between the initial rate of NADH‐O_2_ oxidoreductase activity, reflecting electron transfer from CI to CIV, and the initial rate of TMPD/ascorbate‐O_2_ oxidoreductase activity, reflecting CIV activity alone, following the addition of increasing concentrations of the CIV‐specific inhibitor KCN.

In SHR‐SR cardiac mitochondria, all conditions analyzed exhibited low *C_i_* values (<0.5), indicating that CIV contributed only marginally to flux control and exerted limited influence over total NADH‐O_2_ oxidoreductase activity. Specifically, *C_i_* values were 0.13 in cardiac mitochondria from ND‐fed SHR‐SR rats and 0.35 and 0.26 after 4 and 10 weeks of JD treatment, respectively. In cardiac mitochondria from ND‐fed SHR‐SR rats, KCN titration experiments showed limited control of electron flux through NADH‐O_2_ oxidoreductase activity, with *C_i_* = 0.13 (Figure 2A,B), and the corresponding threshold plot displayed a distinct break point (Figure 2C). This kinetic profile indicated that electron transfer was not efficiently channeled through respiratory SCs. Consistent with these findings, 2D BN/SDS–PAGE revealed the presence of both N‐respirasomes (CI–CIII_2_–CIV*_n_*) and Q‐respirasomes (CIII_2_–CIV*_n_*) (Figure 2D), whereas quantitative analysis of bound and free complexes showed that CIV was predominantly present in free monomeric and dimeric forms (Figure 2E). JD administration did not restore efficient metabolic flux control of aerobic NADH oxidation in SHR‐SR cardiac mitochondria. The low CIV‐dependent control of NADH‐O_2_ oxidoreductase activity was confirmed at both 4 weeks, with *C_i_* = 0.35 (Figure 2F,G), and 10 weeks, with *C_i_* = 0.26 (Figure 2K,L) of JD treatment, and was further supported by the threshold plots shown in Figure 2H,M, respectively. Structural analysis by 2D gel electrophoresis demonstrated that both N‐ and Q‐respirasomes persisted after 4 weeks (Figure 2I) and 10 weeks (Figure 2N) of JD treatment. However, these assemblies were not associated with kinetically efficient channeled NADH flux. Quantitative evaluation of free and bound respiratory complexes again showed a high abundance of free CIV after both 4 weeks (Figure 2J) and 10 weeks (Figure 2O) of JD treatment. Overall, these data showed that CIV, despite being the terminal ETC complex, did not exert effective control over NADH‐dependent respiratory flux in SHR‐SR cardiac mitochondria, irrespective of diet or treatment duration.

**FIGURE 2:**
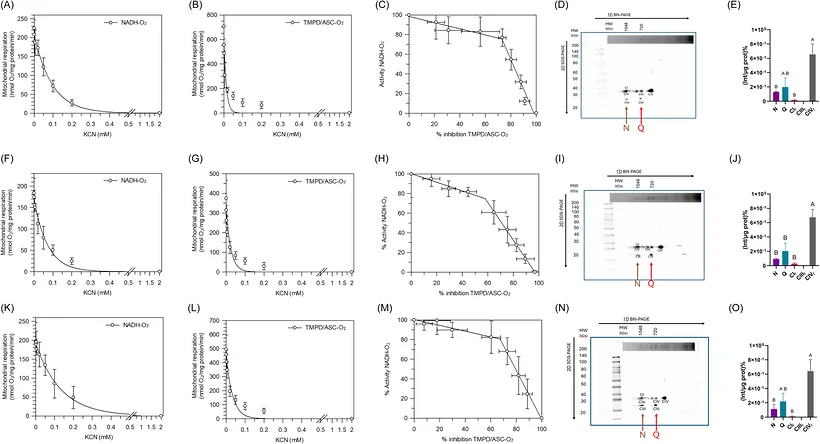
Flux control coefficient of aerobic NADH oxidation controlled by CIV in SHR‐SR heart mitochondria. Inhibitory KCN titration analysis of the respiratory activities in SHR‐SR ND (A–C), JD 4 weeks (F–H), and JD 10 weeks (K–M). Profile of global flux with the stepwise inhibition by KCN of NADH‐O_2_ oxidoreductase activity (A, F, and K) and individual flux with the stepwise inhibition by KCN of TMPD/ASC‐O_2_ oxidoreductase activity (B, G, and L). Threshold plots of NADH oxidase activity (C, H, and M), where each point represents the % activity of aerobic NADH oxidation with CI as a function of % inhibition of TMPD/ASC‐O_2_ oxidoreductase activity with CIV at the same KCN concentration. The respiratory substrates sustaining mitochondrial respiration were 75 µM NADH and 0.5 mM TMPD/2 mM ASC. All points represent the mean ± SD (vertical bars in A, B, F, G, K, and L) and (vertical and horizontal bars in C, H, and M) from at least three independent experiments carried out on different mitochondrial preparations. Supramolecular organization of respiratory complexes in SHR‐SR ND (D), JD 4 weeks (I), and JD 10 weeks (N) heart mitochondria detected by 2D BN/SDS–PAGE. The arrows on 2D SDS–PAGE show the position of the N‐respirasome (N) (CI–CIII_2_–CIV*_n_*) and the Q‐respirasome (Q) (CIII_2_–CIV*_n_*). The antibodies used were against the NDUFA9 (36 kDa) subunit of CI, UQCRFS1 (25 kDa) subunit of CIII, and MTCO1 (35 kDa) subunit of CIV. Quantification and ratio of respiratory CI, CIII_2_, and CIV in SHR‐SR ND (E), JD 4 weeks (J), and JD 10 weeks (O) in N and Q superassembly and in complex free (f) form not involved in SCs. Vertical bars represent the mean ± SD from at least three independent experiments carried out on different mitochondrial preparations. Different letters indicate significantly different values (*p* < 0.05). NADH, nicotinamide adenine dinucleotide; TMPD, *N,N,N′,N*′‐tetramethyl‐*p*‐phenylenediamine dihydrochloride.

### 3.3 Limited Flux Control by Cyt *c* Oxidase Over Aerobic NADH Oxidation in SHR‐SP Heart Mitochondria

Cardiac mitochondria from SHR‐SP rats fed with ND or JD were examined to assess both the kinetic behavior of ETC and the supramolecular organization of its respiratory complexes. Metabolic flux control analysis of aerobic NADH oxidation, evaluated under the control exerted by CIV, showed that CIV contributed only weakly to overall flux control under all experimental conditions analyzed, indicating a limited control of this complex over global NADH‐O_2_ oxidoreductase activity. This pattern was observed in ND‐fed SHR‐SP cardiac mitochondria as well as after 4 and 6 weeks of JD treatment. In ND‐fed SHR‐SP cardiac mitochondria, KCN titration experiments performed with NADH and TMPD/ascorbate, probing CI‐ and CIV‐linked respiration, respectively, generated exponentially decreasing inhibition curves and yielded a *C_i_* value of 0.57 (Figure 3A,B). The corresponding threshold plot displayed a distinct break point (Figure 3C), indicating the lack of efficient channeling during aerobic NADH oxidation. Although 2D BN/SDS–PAGE analysis detected both the N‐respirasome (CI–CIII_2_–CIV*_n_*) and the Q‐respirasome (CIII_2_–CIV*_n_*) assemblies (Figure 3D), quantitative analysis of SC‐associated and free respiratory complexes showed that CIV was largely present in free monomeric and dimeric forms (Figure 3E), indicating that electron transfer was not predominantly supported by a fully assembled channeling‐competent SC organization.

**FIGURE 3:**
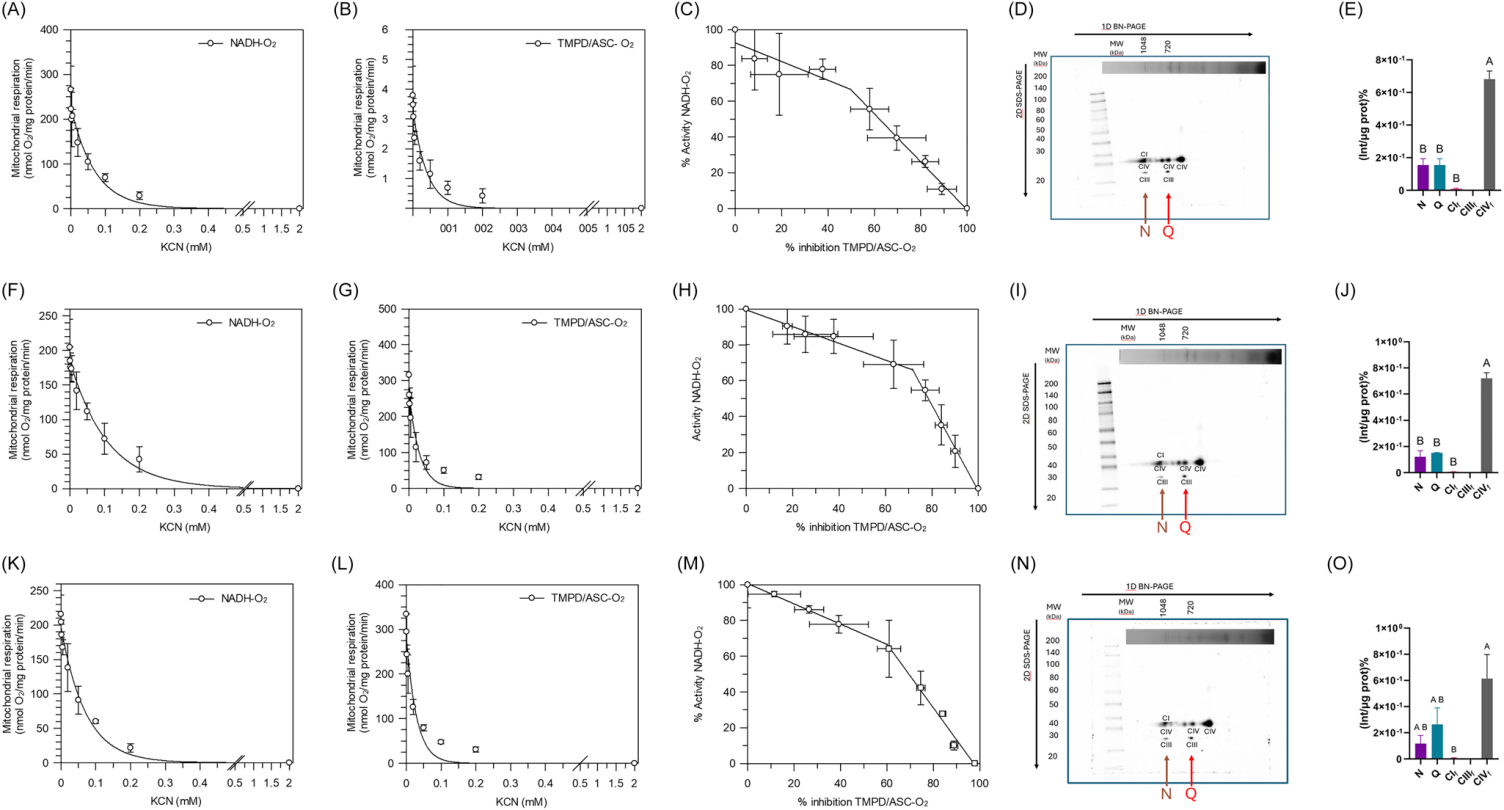
Flux control coefficient of aerobic NADH oxidation controlled by CIV in SHR‐SP heart mitochondria. Inhibitory KCN titration analysis of the respiratory activities in SHR‐SP ND (A–C), JD 4 weeks (F–H), and JD 6 weeks (K–M). Profile of global flux with the stepwise inhibition by KCN of NADH‐O_2_ oxidoreductase activity (A, F, and K) and individual flux with the stepwise inhibition by KCN of TMPD/ASC‐O_2_ oxidoreductase activity (B, G, and L). Threshold plots of NADH oxidase activity (C, H, and M), where each point represents the % activity of aerobic NADH oxidation with CI as a function of % inhibition of TMPD/ASC‐O_2_ oxidoreductase activity with CIV at the same KCN concentration. The respiratory substrates sustaining mitochondrial respiration were 75 µM NADH and 0.5 mM TMPD/2 mM ASC. All points represent the mean ± SD (vertical bars in A, B, F, G, K, and L) and (vertical and horizontal bars in C, H, and M) from at least three independent experiments carried out on different mitochondrial preparations. Supramolecular organization of respiratory complexes in SHR‐SP ND (D), JD 4 weeks (I), and JD 6 weeks (N) heart mitochondria detected by 2D BN/SDS–PAGE. The arrows on 2D SDS–PAGE show the position of the N‐respirasome (N) (CI–CIII_2_–CIV*_n_*) and the Q‐respirasome (Q) (CIII_2_–CIV*_n_*). The antibodies used were against the NDUFA9 (36 kDa) subunit of CI, UQCRFS1 (25 kDa) subunit of CIII, and MTCO1 (35 kDa) subunit of CIV. Quantification and ratio of respiratory CI, CIII_2_, and CIV in SHR‐SP ND (E), JD 4 weeks (J), and JD 6 weeks (O) in N and Q superassembly and in complex free (f) form not involved in SCs. Vertical bars represent the mean ± SD from at least three independent experiments carried out on different mitochondrial preparations. Different letters indicate significantly different values (*p* < 0.05). NADH, nicotinamide adenine dinucleotide; TMPD, *N,N,N′,N*′‐tetramethyl‐*p*‐phenylenediamine dihydrochloride.

After 4 weeks of JD treatment, the *C_i_* value decreased further to 0.27, as calculated from the inhibition curves obtained with NADH and TMPD/ASC (Figure 3F,G). The threshold plot again showed a marked discontinuity (Figure 3H), confirming the absence of efficient metabolic flux control of aerobic NADH oxidation by CIV. Under the same condition, 2D BN/SDS–PAGE still revealed the presence of both N‐ and Q‐respirasomes (Figure 3I), whereas the distribution of free and bound respiratory complexes again demonstrated a high abundance of free CIV (Figure 3J). At 6 weeks of JD treatment, corresponding to the stage at which the stroke phenotype became evident, kinetic analysis continued to show impaired channeling of NADH‐supported electron flux. The *C_i_* value remained low (0.41), as determined from KCN titration curves of NADH‐ and TMPD/ASC‐dependent oxidation (Figure 3K,L), and the threshold plot again exhibited a break in linearity (Figure 3M). Structural analysis by 2D BN/SDS–PAGE confirmed the persistence of both CI–CIII_2_–CIV*_n_* and CIII_2_–CIV*_n_* assemblies (Figure 3N), but quantitative evaluation showed that CIV was still predominantly recovered in the free form (Figure 3O). Overall, SHR‐SP heart mitochondria displayed persistently low CIV‐dependent flux control over aerobic NADH oxidation, and JD treatment was associated with a further deterioration in the kinetics of ETC electron transfer despite the structural maintenance of respiratory SCs.

### 3.4 Exogenous Cyt *c* Supplementation to Restore Cyt *c* Oxidase‑Dependent Flux Control

Flux control analysis of aerobic NADH oxidation in heart mitochondria from SHR‑SR and SHR‑SP rats revealed consistently low control exerted by CIV under all dietary and time‑dependent conditions examined (Figures 2 and 3), with flux control coefficients persistently below 0.5. This kinetic behavior indicated impaired channeling of electron transfer despite the structural detection of both N‑respirasome and Q‑respirasome assemblies by 2D BN/SDS‑PAGE and was associated with a marked accumulation of CIV in a free, non‐SC‑bound form. To test whether the restricted electron flux was due to limited cyt *c* availability, identical kinetic experiments were performed in the presence of excess exogenous cyt *c* (5 µM). Under these conditions, inhibition titrations of NADH‐O_2_ and TMPD/ascorbate‐O_2_ oxidoreductase activities still yielded low *C_i_* values across all groups: 0.24 in SHR‑SR ND at 4 weeks (Figure 4A,B), 0.24 in SHR‑SR JD at 4 weeks (Figure 4D,E), 0.18 in SHR‑SR JD at 10 weeks (Figure 4G,H), 0.27 in SHR‑SP ND at 4 weeks (Figure 4J,K), 0.24 in SHR‑SP JD at 4 weeks (Figure 4M,N), and 0.42 in SHR‑SP JD at 6–8 weeks (Figure 4P,Q). Threshold plots constructed from the percentage of NADH‐O_2_ activity versus the percentage inhibition of TMPD/ascorbate‐O_2_ activity in the presence of increasing KCN concentrations consistently displayed breakage lines in SHR‑SR (Figure 4C,F,I) and SHR‑SP (Figure 4L,O,R) cardiac mitochondria, further documenting the lack of effective channeling. Together, these kinetic results demonstrate that increasing the cyt *c* pool did not restore flux control, indicating that CIV itself remained the rate‑limiting step for NADH oxidation under all tested dietary and pathological conditions.

**FIGURE 4:**
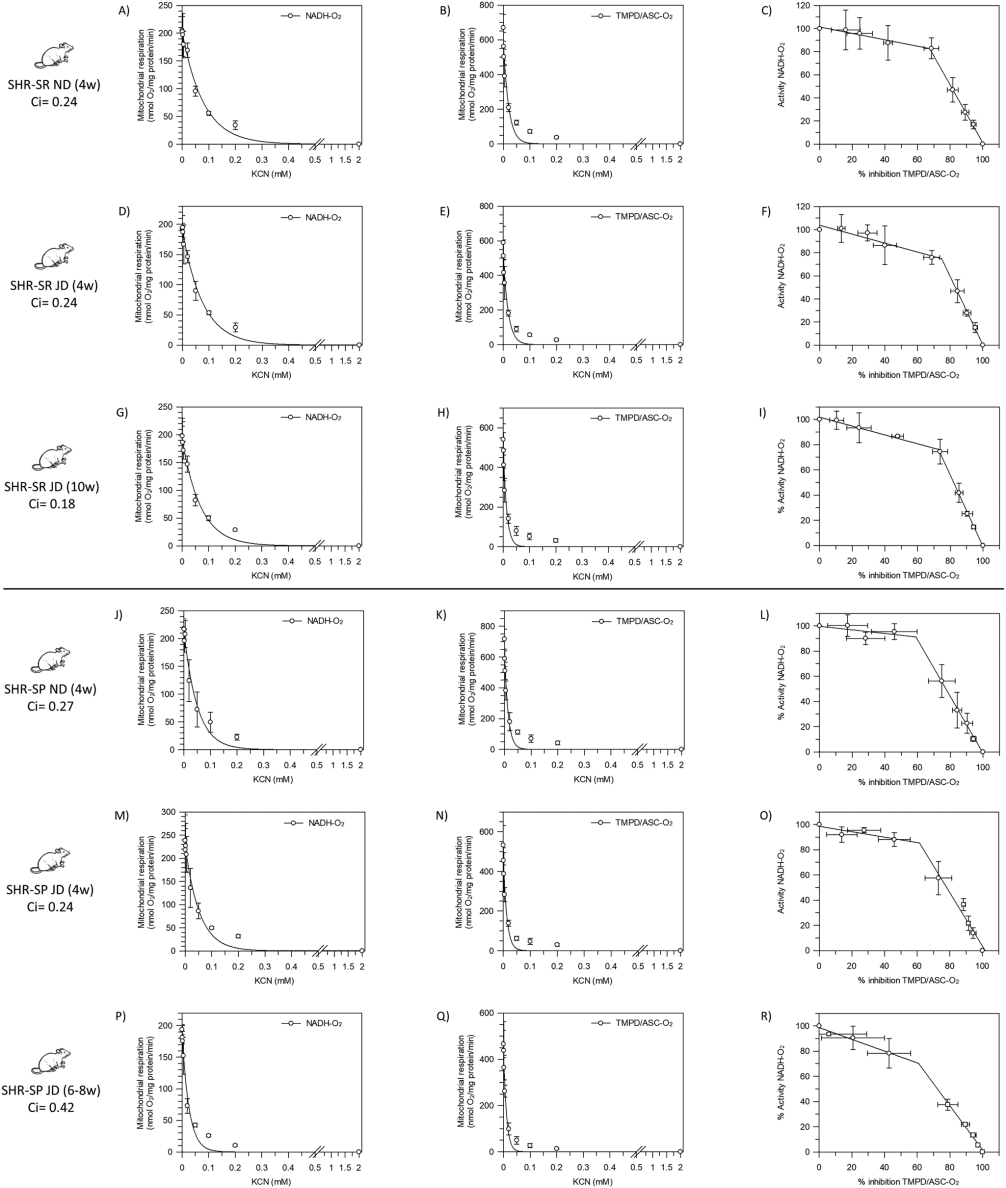
Control exerted by cytochrome *c* oxidase on aerobic NADH oxidation in the presence of exogenous cyt *c*. Inhibitory KCN titration analysis of the respiratory activities in SHR‐SRP ND (A–C), JD 4 weeks (D–F), and JD 10 weeks (G–I) and in SHR‐SP ND (J–L), JD 4 weeks (M–O), and JD 6–8 weeks (P–R) supplemented with 5 µM of cyt *c*. Profile of global flux with the stepwise inhibition by KCN of NADH‐O_2_ oxidoreductase activity (A, D, G, J, M, and P) and individual flux with the stepwise inhibition by KCN of TMPD/ASC‐O_2_ oxidoreductase activity (B, E, H, K, N, and Q). Threshold plots of NADH oxidase activity (C, F, I, L, O, and R), where each point represents the % activity of aerobic NADH oxidation with CI as a function of % inhibition of TMPD/ASC‐O_2_ oxidoreductase activity with CIV at the same KCN concentration. The respiratory substrates sustaining mitochondrial respiration were 75 µM NADH and 0.5 mM TMPD/2 mM ASC. All points represent the mean ± SD (vertical bars in A, B, D, E, G, H, J, K, M, N, P, and Q) and (vertical and horizontal bars in C, F, I, L, O, and R) from at least three independent experiments carried out on different mitochondrial preparations. NADH, nicotinamide adenine dinucleotide; SHR‐SP, stroke‐prone spontaneously hypertensive rat; SHR‐SR, stroke‐resistant spontaneously hypertensive rat; TMPD, *N,N,N′,N*′‐tetramethyl‐*p*‐phenylenediamine dihydrochloride.

### 3.5 CIV Distribution Relative to Complex I and Complex III, and the Excess Capacity of Cyt *c* Oxidase

To determine whether the predominance of free CIV reflects a CIV‐specific dissociation from N‐ and Q‐respirosomes, we quantified the free (non‐SC‐associated) fraction of CI and CIV as a proportion of each complex's total abundance. Complex III could not be included in this comparison because no CIII signal was detected outside the N‐ and Q‐respirosome bands. Across all SHR‐SR and SHR‐SP groups, irrespective of diet or duration, CI was almost entirely confined to supramolecular assemblies, with only a minimal free fraction (0.5%–2.9% of total CI; Table 1). In marked contrast, CIV remained free in 61%–72% of its total pool across the same groups (Table 1). This large disparity indicates that the predominance of free CIV is not a generic feature of respiratory‐complex distribution in SHR heart mitochondria, but a property specific to CIV, and is consistent with CI distribution being retained within N‐ and Q‐respirosomes far more efficiently than CIV.

**TABLE 1:** CIV/(CI + CIII) total ratio stoichiometry, CIV excess capacity, and *C_i_* across experimental groups.

Group	$J_{O_{2}}$ NADH‐O_2_ (nmol O_2_/mg/min)	$J_{O_{2}}$ TMPD/ASC‐O_2_ (nmol O_2_/mg/min)	CIV excess capacity (a.u.)	CIV free (% of total CIV)	CI free (% of total CI)	CIV/(CI + CIII) total ratio (a.u.)	*C_i_* (a.u.)
SHR‐SR ND 4w	224.8	703.1	3.13	65.5	1.6	48.7	0.13
SHR‐SR JD 4w	184.0	375.0	2.04	67.4	2.9	26.6	0.35
SHR‐SR JD 10w	196.7	492.5	2.50	64.4	1.0	100.1	0.26
SHR‐SP ND 4w	265.9	379.2	1.43	68.2	0.9	94.0	0.57
SHR‐SP JD 4w	204.4	315.6	1.54	72.0	1.0	113.1	0.27
SHR‐SP JD 6–8w	215.8	334.0	1.55	61.4	0.5	165.3	0.41
WKY (normotensive)^a^	145.8	236.4	1.62	71.7	0.50	146.0	0.17

*Notes*: Values are mean ± SD. CIV free and CI free indicate the percentage of each complex not incorporated into N‐ or Q‐respirosomes, determined by 2D BN/SDS–PAGE.

^a^ WKY values were calculated from the oxygraphic and proteomic dataset underlying Glogowski et al. [25] (*C_i_* as reported therein), using the same calculation procedures applied to the SHR groups; WKY values are included for reference only and were not tested for statistical significance against the SHR groups.

Abbreviations: JD, Japanese diet; NADH, nicotinamide adenine dinucleotide; ND, normosodic diet; SD, standard deviation; SHR‐SP, stroke‐prone spontaneously hypertensive rat; SHR‐SR, stroke‐resistant spontaneously hypertensive rat; TMPD, *N*,*N*,*N*′,*N*′‐tetramethyl‐*p*‐phenylenediamine dihydrochloride; WKY, Wistar–Kyoto.

To determine whether this reflects an increase in the total abundance of CIV relative to CI and CIII, rather than only a redistribution between free and SC‐bound pools, we additionally calculated the ratio of total CIV signal (free plus N‐ and Q‐incorporated) to the combined total CI and CIII signal for each group, using absolute densitometric intensities (int/µg protein) from the same proteomic dataset. This total CIV/(CI + CIII) ratio (Table 1) showed considerable within‐group variability (coefficient of variation frequently exceeding 50%) and no consistent strain‐ or diet‐dependent pattern. Taken together with the free‐fraction data above, these results indicate that the predominance of free CIV in SHR heart mitochondria reflects a redistribution of CIV between free and SC‐associated pools, rather than a net increase in total CIV abundance relative to CI and CIII.

As summarized in Table 1, CIV excess capacity was consistently higher in SHR‐SR cardiac mitochondria (2.04‐ to 3.13‐fold across ND and JD conditions) than in SHR‐SP cardiac mitochondria (1.43‐ to 1.55‐fold). This narrower margin between CIV capacity and upstream flux demand in SHR‐SP cardiac mitochondria paralleled the higher *C_i_* values already reported for this strain, indicating that CIV operates closer to its rate‐limiting threshold in SHR‐SP than in SHR‐SR heart mitochondria. To contextualize these findings against a non‐hypertensive reference, we compared them with data recently obtained using an experimental protocol identical to that used here (same mitochondrial isolation procedure, Clark‐type oxygraphy, NADH or TMPD/ASC substrates, KCN titration, and *C_i_* calculation), in heart mitochondria from normotensive Wistar–Kyoto (WKY) rats [25]. In that study, WKY hearts displayed a flux control coefficient of *C_i_*  =  0.17 and a predominance of free, non‐SC‐associated CIV despite the detectable presence of N‐ and Q‐respirosomes. Applying the same calculation procedures used for the SHR groups to the oxygraphic and proteomic dataset underlying that study, WKY hearts showed $J_{O_{2}}$ NADH‐O_2_ = 145.8 and $J_{O_{2}}$ TMPD/ASC‐O_2_ = 236.4 nmol O_2_/mg/min, corresponding to a CIV excess capacity of 1.62 a.u., together with 71.7% of CIV and only 0.50% of CI in free, non‐SC‐associated form (Table 1). Notably, the WKY excess capacity value (1.62) falls close to the range observed in SHR‐SP cardiac mitochondria (1.43–1.55) and below that of SHR‐SR cardiac mitochondria (2.04–3.13), a pattern that, given the single normotensive reference group available, should be interpreted with caution as the WKY study was not designed as a formal statistical comparator to the SHR strains, and as the WKY data derive from a single experimental group rather than the multiple dietary/temporal cohorts available for SHR‐SR and SHR‐SP, we cannot report a direct significance test between groups. However, the correspondence between these independently obtained, methodologically identical datasets indicate that the low *C_i_* values and the predominance of free CIV described in the present hypertensive model are not an artifact of the experimental approach but reflect a kinetic and structural organization of the terminal oxidase segment that is already present under basal, non‐pathological conditions, and that becomes progressively more constrained (lower excess capacity, higher *C_i_*) in the stroke‐susceptible SHR‐SP strain.

## 4 Discussion

The present study provides convergent kinetic and structural evidence that, in cardiac mitochondria from both SHR‐SR and SHR‐SP rats, the supramolecular detection of respiratory SCs is not sufficient, per se, to ensure efficient channeling of NADH‐derived electron flux. Across all experimental conditions examined (Figures 2 and 3), flux control analysis consistently assigned a limited control coefficient to CIV, with *C_i_* values generally remaining below the threshold expected for tightly channeled electron transfer. This finding is mechanistically relevant because it reveals a clear dissociation between the structural persistence of N‐ and Q‐respirasomes and the functional preservation of optimized respiratory flux [24]. Such a dissociation is consistent with the current view that respiratory SCs are dynamic entities whose mere presence does not necessarily imply maximal catalytic coupling, as their functional competence depends on multiple parameters, including stoichiometry, membrane lipid environment, redox‐dependent organization, and the relative abundance of free versus SC‐bound complexes [26, 27].

A central outcome of the present work is the identification of CIV as a poorly integrated terminal step in the pathway of aerobic NADH oxidation, despite its structural detection within SC‐containing fractions. In both SHR‐SR and SHR‐SP cardiac mitochondria, quantitative analyses revealed a marked enrichment of CIV in free, non‐SC‐associated forms. This observation provides a plausible explanation for the kinetic phenotype observed. If a substantial fraction of CIV is not effectively incorporated into a functionally competent CI–CIII_2_–CIV axis, electron transfer from NADH oxidation is expected to proceed through a less organized, diffusion‐mediated regime, resulting in a loss of effective channeling and the appearance of threshold plots with clear discontinuities independently of condition dependent of time and diet for SHR‐SR (Figure 2C,H,M) or SHR‐SP (Figure 3C,H,M) rats. In this context, the low *Ci* values observed here strongly suggest that electron transfer from CI to CIV does not predominantly occur through a tightly integrated supramolecular pathway, but rather through weaker and less constrained interactions between mobile carriers and individual respiratory complexes [12, 28].

This interpretation is further supported by quantification of CIV excess capacity and CIV/CI relative stoichiometry (Table 1), which were consistently narrower and more asymmetric, respectively, in SHR‐SP than in SHR‐SR cardiac mitochondria. Within the framework of metabolic control analysis, a smaller margin between CIV capacity and upstream flux demand is expected to shift control toward the terminal oxidase, as directly demonstrated by KCN titration studies in human skeletal muscle, where a reduction in COX excess capacity from 2.3‐ to 1.4‐fold was accompanied by a corresponding increase in the flux control coefficient from 0.27 to 0.36 [29]. We propose that the reduced CIV reserve capacity observed in mitochondria of SHR‐SP hearts may represent a functional correlate of the more severe phenotype (stroke susceptibility) characterizing this strain, warranting further investigation as a candidate contributor to disease‐associated mitochondrial vulnerability. Notably, comparable *C_i_* values and a similar predominance of free, non‐SC‐associated CIV have recently been reported in normotensive WKY rat heart mitochondria analyzed under an experimental protocol identical to that used here (*C_i_* = 0.17) [25], indicating that this kinetic behavior—low flux control by CIV under basal, non‐hypertensive conditions, shifting toward markedly higher control in the SP SHR‐SP strain—is already detectable in a normotensive reference and is not an artifact of the methodological approach used in the present study. Moreover, the CI/CIV asymmetry of WKY closely parallels the pattern observed in SHR‐SR and SHR‐SP cardiac mitochondria, indicating that the predominance of free, kinetically rate‐limiting CIV is a feature of the basal cardiac respiratory chain and not specific to the hypertensive strains [25, 30].

Thus, the limiting factor in SHR heart mitochondria does not appear to be the absence of respirasome assemblies, but rather their incomplete functional integration, likely due to the predominance of CIV outside the channeling‐competent fraction [31, 32]. These data also refine the interpretation of SCs function in hypertensive cardiac mitochondria. Although SCs have been proposed to stabilize CI, optimize electron transfer, and limit electron leakage under selected conditions, their impact appears to be strongly context‐dependent rather than absolute [16]. In the present setting, the structural maintenance of N‐ and Q‐respirasome assemblies did not translate into effective CIV‐dependent control of NADH‐supported respiration. This indicates that the existence of SC bands on 2D BN/SDS–PAGE should not be equated with full kinetic competence [33]. Instead, channeling efficiency appears to depend on whether CIV is quantitatively and functionally integrated into a productive electron‐transfer route. Therefore, the present findings support the view that ETC organization must be evaluated not only at the structural level, but also through direct kinetic analysis, as structural preservation alone may mask profound defects in respiratory flux control [30, 34].

A further implication of the present findings concerns mitochondrial redox balance. SC‐dependent electron channeling has been proposed to limit ROS formation not merely as a structural byproduct, but as a kinetic consequence of minimizing the residence time of electrons at upstream redox centers [27]. When CIV is predominantly excluded from a functionally competent CI–CIII–CIV route, as observed in this study, electrons entering the chain from NADH oxidation are expected to accumulate upstream of the terminal oxidase, favoring over‐reduction of CI and CIII and thereby increasing the probability of electron leakage to oxygen at these established ROS‐generating sites [35]. ROS formation was not directly assessed in the present samples because reliable ROS quantification requires freshly isolated, actively respiring mitochondria, whereas the specimens used here were processed as snap‐frozen cardiac tissue for the polarographic and proteomic analyses described above; however, the disrupted CI‐to‐CIV channeling, the predominance of free CIV, and its progressively narrower excess capacity described here are consistent with a redox environment permissive for increased mitochondrial ROS production [17], particularly in SHR‐SP hearts, where CIV excess capacity is most severely reduced (Table 1). This possibility warrants direct investigation in future studies using freshly isolated mitochondria from this model.

An additional mechanistic insight derives from the experiments performed in the presence of excess exogenous cyt *c*. These analyses were specifically designed to test whether the impaired CIV‐dependent flux control could be explained by limited availability or restricted mobility of the soluble electron carrier within the intermembrane space and between free respiratory complexes or SCs. However, increasing the cyt *c* pool failed to restore high *C_i_* values in any of the groups analyzed (Figure 4), and threshold plots continued to display the characteristic break points indicative of non‐channeled electron transfer. These results argue against a simple carrier‐limitation model and instead indicate that the primary defect resides at the level of CIV integration or in the kinetic organization of the CIII–CIV segment. Indeed, although cyt *c* can support electron transfer by diffusion along the surface of CIII–CIV assemblies, this process requires a properly configured topological and functional interface. Accordingly, increasing the bulk cyt *c* pool cannot compensate for an unfavorable organization in which CIV is only partially incorporated into a channeling‐competent supramolecular pathway. Under these conditions, CIV itself remains the rate‐limiting step of NADH oxidation [36, 37].

A relevant mechanistic implication of these findings concerns the possible contribution of SCAF1 and NDUFC2 to the defective kinetic integration of the respiratory chain in hypertensive myocardium. SCAF1 promotes the formation of CI–CIII_2_–CIV and CIII_2_–CIV assemblies and supports respiratory efficiency, but its presence does not necessarily guarantee effective channeling when CIV remains poorly incorporated into functional SCs [15, 38]. In parallel, NDUFC2 deficiency has been directly linked to CI dysfunction, mitochondrial damage, stroke susceptibility in SHRSP rats, and cardiac hypertrophy in human hypertension [5, 39]. Thus, altered SCAF1‐dependent SC organization together with impaired NDUFC2‐dependent CI stability may contribute to the CIV‐centered bottleneck observed here.

The pathophysiological implications of these findings are particularly relevant in the context of hypertensive disease progression and stroke susceptibility. In both SHR‐SR and SHR‐SP rats, the kinetic defect in CIV‐dependent flux control was observed independently of significant differences in systolic blood pressure, proteinuria, or body weight during the pre‐stroke observation period (Figure 1). This indicates that the mitochondrial phenotype described here cannot be simply attributed to overt systemic deterioration but rather suggests that ETC dysfunction may represent an early and relatively autonomous feature of the hypertensive state. In this regard, the persistence of low CIV‐dependent flux control in hypertension supports the idea that defective kinetic organization of NADH oxidation is embedded within the hypertensive mitochondrial background and may contribute to the increased vulnerability to stroke. This interpretation is in agreement with growing evidence that mitochondrial dysfunction is an early determinant of cardiovascular and cerebrovascular injury, and with recent data indicating that preservation of respiratory complex organization and SC integrity is associated with protection from stroke development in SHR‐SR relative to SHR‐SP animals [24, 40, 41].

Collectively, the present findings highlight a critical functional uncoupling between ETC architecture and respiratory kinetics in hypertensive heart mitochondria. Although respiratory SCs remained structurally detectable in all experimental groups, they were unable to support effective CIV‐dependent flux control of aerobic NADH oxidation. The data therefore support a model in which cardiac mitochondrial respiration in SHR rats is constrained by a CIV‐centered kinetic bottleneck that is not corrected by JD exposure and is not rescued by exogenous cyt *c* supplementation. The excess of free CIV likely reflects a dispersed terminal oxidase pool that is structurally preserved but kinetically suboptimal, thereby uncoupling SC assembly from effective channeling of electron flux. More broadly, these observations indicate that therapeutic strategies aimed solely at preserving or enhancing SC assembly may be insufficient unless they also restore proper CIV integration and functional control within the ETC. In this respect, the combined structural and kinetic approach adopted here provides a more accurate framework for interpreting mitochondrial respiratory efficiency in hypertensive cardiac pathology.

## Author Contributions

**Patrycja Anna Glogowski**: formal analysis, validation, writing – original draft. **Cristina Algieri**: formal analysis, validation. **Antonia Cugliari**: formal analysis, validation. **Maria Cotugno**: investigation, methodology. **Fabiana Trombetti**: writing – review and editing. **Micaela Fabbri**: methodology. **Arrigo Francesco Giuseppe Cicero**: resources. **Speranza Rubattu**: conceptualization, funding acquisition. **Salvatore Nesci**: conceptualization, validation, visualization, supervision, funding acquisition, writing – original draft.

## Funding

The research leading to these results received funding from European Union—Next Generation EU, Missione 4 Componente 1 CUP J53D23008970006, Progetto PRIN 2022 UNDER40 (no. 2022E75TWB_001) to Speranza Rubattu and Salvatore Nesci.

## Conflicts of Interest

The authors have nothing to report.

## Data Availability

Data are available on AMSActa Institutional Research Repository by AlmaDL University of Bologna Digital Library https://doi.org/10.6092/unibo/amsacta/8938.
